# A latent growth curve modelling approach to seasonal and spatial dynamics of food security heterogeneities in rural Lake Naivasha Basin, Kenya

**DOI:** 10.1007/s12571-021-01200-9

**Published:** 2021-07-17

**Authors:** Maria Sassi, Gopal Trital

**Affiliations:** Department of Economics and Management, University of Pavia, Via S. Felice, 5-27100, Pavia, Italy

**Keywords:** Latent growth curve model, Household food security, Seasonal dynamics, Plantation sector, Subsistence agriculture, Kenya

## Abstract

The increasing complexity of food insecurity, malnutrition, and chronic poverty faced by Sub-Saharan Africa warrants urgent categorisation and tracking of household food security along both temporal and spatial dimensions. This will help to effectively target, monitor and evaluate population-level programs and specific interventions aimed at addressing food insecurity. Traditional longitudinal analysis does not address the dynamics of inter- and intrahousehold heterogeneities within the seasonal and spatial context of household-level food security. This study is the first to overcome such limitations by adopting a multi-group piecewise latent growth curve model in the analysis of the food security situation in a statistically representative sample of 601 households involved in subsistence and cut-flower commercial agriculture, around Lake Naivasha. We considered food security as a latent concept, which manifests as food security outcomes in our primary longitudinal dataset from March 2018 to January 2019. Our analysis highlights the temporal and spatial dynamics of food security and advances new evidence on inter- and intrahousehold heterogeneities in food security across different seasons for the subsistence and commercial farming clusters. These heterogeneities were demonstrated primarily during the hunger season from March to June, and persisted in both the clusters and across months, albeit with different intensities. Moreover, our results indicate the importance of commercial agriculture in achieving food security in the hunger season. Our study suggests the need of a multidisciplinary approach to food security and the introduction of well-coordinated interventions for the development of subsistence and commercial agriculture considering the seasonal and cluster-level specificities.

## Introduction

The interrelated challenges of food security, malnutrition, and chronic poverty are becoming more complex in the current global scenario, especially for developing countries in Sub-Saharan Africa (FAO et al., 2020). A desert locust outbreak in the Horn of Africa and the COVID-19 pandemic in these countries involving a fragile and informal economy, dominant subsistence agricultural sector and weak healthcare systems, are undermining sustainable livelihoods and food and nutrition security for many already poor and vulnerable people (Chiwona-Karltun et al., 2021; Devereux et al., 2020; FAO, 2020; Uwaezuoke, 2020). This situation warrants urgent categorisation and tracking of household food security to support effective targeting, monitoring, and evaluation of population-level programs and specific interventions aimed at addressing food insecurity (Webb et al., 2006).

The empirical literature highlights seasonality and vulnerability as important factors shaping the dynamics of food security in Sub-Saharan Africa (Devereux et al., 2013; Sassi, 2015, 2019). The region witnesses substantial seasonal and spatial variation in crop production and household-level food access conditions (Bolarinwa et al., 2020; Brander et al., 2021; Cedrez et al., 2020; Sassi, 2019; Thornton et al., 2009). Consequently, an understanding of both temporal and spatial dimensions of household food security status should be important for better designing the targeting of food security interventions.

Knowledge of dietary diversity is one of the elements currently used to inform food security analysis employed in the design and targeting of interventions by organisations such as the WFP and FAO. In particular, the Food Consumption Score (FCS) has gained popularity as a composite measure of food frequency, dietary diversity, and the relative nutritional importance of different food groups at the household level (Marivoet et al., 2019; Sassi, 2018). FCS provides a snapshot of current food consumption, which then forms a basis for the categorisation of households into three food secure/insecure groups, namely poor, borderline and acceptable (World Food Programme, Vulnerability Analysis and Mapping Branch, 2008). Moreover, it is a standardised measure, and therefore, can be adopted for comparisons of the households’ state of food security over time and locations. However, a classification of households into categories of food secure and insecure groups does not completely capture the population-level dynamics of changing food security levels and status across time. For example, the fact that the prevalence of food insecurity has increased over time does not explain the rate or any differential effect of such a change on the food security status of households. We can find a situation in which, despite an overall increase in the prevalence of food insecurity in the population, a group of households might witness an improvement in their food security levels. The understanding of these circumstances and any meaningful generalisation to the reference population have important operational implications and, thus, require a more rigorous longitudinal analysis.

Traditionally, the longitudinal analysis of food security is limited to understanding the mean-level changes in the food security indicators for an average household over time (Aurino et al., 2020; Mutisya et al., 2016). Such an approach assumes that the rate of change in food security outcomes is applicable uniformly for all the households across the population. As a consequence, general conclusions made for an average household might guide the design of policy interventions, however, overlooking any time-specific changes in the inter- and intrahousehold heterogeneities of household-level food security levels. The limitation of traditional longitudinal analysis has led to an increased application of growth curve models in development research as they capture both inter and intrahousehold heterogeneity in the change of outcome measures over time (Bollen & Curran, 2006; McArdle, 2009; Preacher, 2008). Nevertheless, the application of growth curve models is very limited in household-level food security research, and there exists a literature gap in understanding the dynamics of interhousehold differences in the intrahousehold changes of food security outcomes over time, particularly in the local context of Kenya. The temporal and spatial analysis of food security is often hampered by the lack of household-level data across time and space (Fraval et al., 2019). We cover this gap by adopting the latent growth curve modelling (GCM) approach in our analysis capitalizing on the monthly household-level panel data that we collected in the semi-arid middle and lower catchment of the Lake Naivasha Basin in Nakuru County in the south-eastern part of the Rift Valley Province, Kenya.

Lake Naivasha region is the hub of Kenya’s cut flower industry that generates over 50% of the country’s total flower production (Bolo, 2006). The selection of our sample was motivated by the specific features of the area surrounding Lake Naivasha Basin where a small-scale subsistence-based farming sector coexists with a flower enclave that exhibits the characteristics of cash-crop plantations as indicated by Smalley (2013, p. 7). In line with these dominant features, we distinguished two exclusive clusters in our research area: a subsistence-agricultural cluster that refers to the areas with a prominent subsistence and rain-fed sector, and the plantation cluster for zones with strong presence of the cut-flower plantation sector.

Our sample comprised the rural households in these two clusters. As highlighted in Sassi (2019), rural households face different vulnerabilities to food security in these two typologies of clusters, and seasonality stands as a critical dimension affecting their food and nutrition security. This situation allowed us to test whether the latent growth process of food security is cluster-specific and if such a process favours the characteristics of the market-based commercial food system being promoted in East Africa (Pinstrup-Andersen, 2013; Sassi, 2019). In Kenya, the government and subsequently the Nakuru County where the investigated area is located, have adopted food security and agricultural development policies highlighting the important role of agriculture for the eradication of food insecurity and poverty (Republic of Kenya, 2007, 2011, 2013, 2016). Such policy supports a new wave of capital accumulation in agricultural development, however, undermining the role of plantations and estates where large-scale commercial agriculture is already in place (Goldsmith, 2016). For example, the Agricultural Sector Development Strategy as included in the policy aims to achieve a paradigm shift from subsistence to commercial agriculture. The Strategy document envisions the realization of this objective through the transformation of smallholder agriculture from subsistence to an innovative, commercially-orientated and modern agricultural sector (Republic of Kenya, 2011). However, the Strategy does not refer to plantations and estates and their role in the achievement of food security. We have therefore analyzed the role of the plantation cluster for the improvement of food security in comparison to the dominant smallholder farming sector to provide an understanding of how large-scale commercial agricultural models affect the food security of rural households. This information is relevant for planning food and nutrition security interventions coherent with Goal 2 of the Sustainable Development Goals (United Nations, 2018) in the rural-Kenyan context.

Our paper acknowledges that food security is a latent concept as also identified in the literature (Vaitla et al., 2017). With that consideration, we analyze food security as the underlying growth process using growth curve models (GCM). Our methodological approach recognizes that the underlying growth process of food security itself is unobservable, but manifests itself as food security outcomes at each unit of time as measured by FCS. While the adoption of growth curve models (GCM) in the analysis of repeated food security measures can follow several methodological approaches, including that of a multi-level modelling framework (Bollen & Curran, 2006), we adopted the latent GCM approach within the Structural Equation Modelling (SEM) framework in our analysis. Our study is the first to have adopted the SEM-based growth curve modelling approach in investigating the food security situation over time with a comprehensive exploration of inter- and intrahousehold heterogeneities. SEM offers robust estimation methods in analysing the growth trajectory of a latent concept of food security in contrast to other competing methods. Besides, SEM also offers flexibility in exploring the effects of time-variant and time-invariant predictors as well as in comparing growth models based on an extensive number of fit-indices towards a robust model estimation (Bollen & Curran, 2006; Whiteman & Mroczek, 2007).

We further extended our growth curve model as a piecewise latent trajectory model (PLTM) to account for the seasonality of the food security situation in our research area. PLTM, or simply piecewise GCM, is a parsimonious model to investigate the dynamics of inter- and intrahousehold non-linear changes as well as in exploring and comparing such changes before and after the specified time points (Duncan et al., 2013; Flora, 2008; Sayer & Willett, 1998). Our paper thus incorporates both temporal and spatial dimensions in the analysis of food security, and therefore, provides evidence to highlight the importance of considering seasonal dynamics and patterns of food security outcomes in the design of targeted policy interventions.

We have also explained our quantitative results with the qualitative evidence that we collected through focused group discussions (FGDs) in each of the clusters of our research area. The FGDs provided more context-specific first-hand description on seasonal patterns of crop production, harvesting and food consumption for our sampled households.

We outline this paper as follows. Section 2 explains our longitudinal data, empirical strategy, and estimation methods; Section 3 presents our results followed by their discussion in Section 4; and Section 5 concludes our paper by highlighting the prospective policy implication of our results along with directions for further research on the topic.

## Materials and methods

### Data and sample

The analysis in this paper refers to the longitudinal data that we collected during a project aimed at studying the state of rural households’ food security and its determinants in Lake Naivasha Basin, Kenya. We used a subset of data gathered every month from March 2018 to January 2019 for a longitudinal sample of 601 rural households from a total population of 28,939 households identified through random sampling methods with the support of the Kenya National Bureau of Statistics (KNBS). Our dataset captures different crop and climatic seasons as illustrated in Table 1.

**Table 1:**
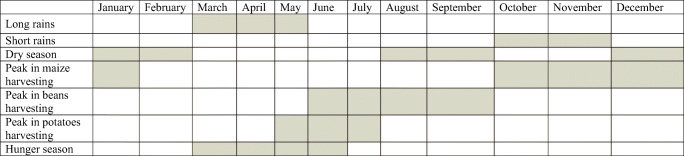
Seasonal and crop calendar. Source: Authors’ elaboration based on primary sources of data.

We paid specific attention to the sample selection process. We used the seven clusters adopted by KNBS in our study area and selected our sample according to the master frame used in the fifth National Sample Survey and Evaluation Program (KNBS, 2014). With the support of local informants and the help of satellite maps, we classified the sampled clusters into floricultural and smallholder agricultural clusters in our analysis according to the dominant influence of floriculture or smallholder farms. The KNBS of Nakuru supported the process of household mapping.

We calculated the longitudinal sample size (*n*) using the Cochran (1997)‘s formula with further correction for the statistical representation of the finite population at the cluster level, as in the following,

1 $$ n=\frac{\frac{z_{\alpha /2}^2}{t^2}{S}^2}{1+\frac{1}{N}\frac{z_{\alpha /2}^2}{t^2}{S}^2} $$

where *z* is the critical value of the desired confidence level, *t* is the desired margin of error, and *S*^2^ is the variance of the sample. We used 1.96 for *z,* corresponding to the confidence level of 95%. In other words, we accepted a 5% probability of type I error (*α*) in our estimates. We have fixed the margin of error in our estimation (*t*) at 4%.

According to this formula, our sample size should include 588 households. We increased the number of households to 606 to avoid a possible reduction of the sample below the representative level due to possible mistakes during data collection. However, we had no drop-out of respondents. Nevertheless, we limited the sample to 601 households in our study based on the data quality and consistency over the investigated period. Fifty-one % of the sampled households were in the plantation cluster and 49% in the agricultural cluster. In both the clusters, household heads were, on average, 48 years old, around 35% of them were illiterate, and around 30% female. The household size was on average two, with a minimum of one and a maximum of five members.

We followed the steps suggested by Cochran (1997) to design our questionnaire aimed at capturing the food and livelihood security of rural households in the study area. We validated the questionnaire in consultation with stakeholders from the National Drought Monitoring Authority of Kenya, National Bureau of Statistics in the Nakuru County Office, Ministry of Agriculture Naivasha Sub-county Office, university staff, and a group of enumerators. An expert linguist translated the questionnaire into Kiswahili and Kikuyu, the two most important vernacular languages in the area.

The questionnaire was submitted by a group of enumerators selected among the staff of the KNBS of Nakuru and students of Nairobi University. We provided training for the enumerators and piloted the questionnaire before the monthly data collection. The questionnaire was submitted through the ODK tool.

A large part of the literature measure household food security referring to the concept of dietary diversification (Islam et al., 2018; Megersa et al., 2014; Romeo et al., 2016). On the contrary, we adopted the Food Consumption Score (FCS) as the observed measure of food security in our analysis. Therefore, our analysis uses a multidimensional indicator and adds to the literature based on dietary diversity alone. However, we acknowledge that FCS is only a proxy indicator of household-level caloric availability and might not perfectly capture the comprehensive dimensions of the food security situation. Nonetheless, FCS provides a quantitative measure of food consumption accounting for dietary diversity and nutrient density at the household level, and thus, qualifies as a key indicator to investigate the underlying food security situation in our research area.

We calculated the FCS for each household in our sample as per the World Food Programme, Vulnerability Analysis and Mapping Branch (2008) protocol in the following steps. First, we grouped all the food items into the nine standard food groups: staple, pulses, vegetables, fruits, meat and fish, milk, sugar, oil, and condiments where each of these groups includes food items with similar caloric and nutrient content. Afterwards, within each of these groups, we summed up the consumption frequencies of all food items consumed during the last seven days preceding the survey for each household. We further fixed the upper limit of the total consumption frequencies as ‘7’ for any values exceeding seven in each food group. Finally, we calculated the household FCS by multiplying the total consumption frequencies obtained for each food group by the standard weights set at 2 for staple, 3 for pulses, 1 for vegetables and fruit, 4 for meat and fish and milk, 0.5 for sugar and oil and 0 for condiments. These standard weights account for the nutrient density of each food group, thus, rendering FCS as a composite measure for both household dietary diversity and nutrient density. Table 5 presents the descriptive statistics of the FCS for the overall sample and the clusters.

We conducted our analysis by grouping the seven counties in our investigated area as the plantation and subsistence-agricultural clusters based on the inherent economic features in each of these counties. The plantation cluster around Lake Naivasha is the hub of Kenya’s cut-flower industry. This area produces 70% of the country’s floriculture production, a sector that overall contributes to almost 1.3% of the country’s gross domestic product (Kirigia et al., 2016). The households in the plantation cluster exhibit the ‘plantation model of farming’ with the following five key characteristics, as also described by Smalley (2013, p. 7). First, the farming practices in this cluster are capital intensive. Second, most farmers grow one main cash crop, which in our sample is mostly cut-flowers. Third, these cash crops are grown on a land size larger than an average-sized holding with parts of land that are left uncultivated. Fourth, the landowners centrally manage farms, and lastly, farming mostly relies on hired resident or non-resident labour. Another important feature is that the plantation cluster, with the characteristics of an enclave, includes not only farmers but also other key actors of the cut-flower industry. These include research institutions, breeding farms, quality control and regulatory agencies, input suppliers, credit and finance institutions, trade promotion agencies, and other intermediary organisations.

On the contrary, the subsistence-agricultural cluster features a rain-fed agricultural system with farms mostly located in water-scarce areas with frequent crop failure. Moreover, the farmers in this cluster are mostly smallholders practising small-scale farming, and they predominantly produce for their subsistence needs. These farmers also engage in selling their excess agricultural produce. That, however, is mostly observed only during the post-harvest period for maize.

Despite these dominant features, we noted a certain degree of variation in the occupational choice of the household head, even within these clusters in our sample. The occupational choice of household head proxies the household-level economic structure as well as other unobserved factors contributing to such choice, which might have an impact on the food security situation of the household at a given month. With the omission of this distinguishing feature of household, we risk a biased conclusion about food security growth trajectories associated with cluster-specific economic features in our research area. Therefore, we introduced a control variable called ‘Occupation’, which equals to 1 for a household head employed in small-scale farming and 0 for floriculture or other sectors.

To further triangulate our results, we also gathered qualitative evidence with the implementation of focus group discussions (FGD) and semi-structured key-informant interviews in each of the seven clusters. Each FGD consisted of eight participants. We conducted the FGDs in accordance with the recommendations made by the literature (Dawson et al., 1993; Mancini Billson, 2006), thereby controlling for gender and age of participants as well as ensuring a balanced number of young and elderly women and men. The composition of FGDs was also made with the consideration of local cultural specificities. In addition to FGDs, we conducted semi-structured interviews with the local community leaders in each of the counties to further enrich our cultural and economic understanding of the evidence collected in the two clusters.

### Empirical strategy

In our analysis, we used an econometric technique that refers to the growth curve models (GCM) family. The core idea of the GCM approach is the estimation of smoothed latent growth trajectories of food security for each household in the sample, as indicated by the repeated measures of FCS observed across months, with the acknowledgement that the individual-specific trajectories are heterogeneous. The average of the individual-specific growth trajectories indicates the overall underlying trajectory of food security and the variability of the trajectories reflect the heterogeneity in the growth process witnessed by the households in our research area. In other words, GCM serves the estimation of the following three typologies of change in the latent process of food security in our research area: (i) ‘intrahousehold growth’ that refers to the rate of change in the overall trajectory of the food security situation over the months; (ii) ‘interhousehold difference’ that captures the differences in the food security situation between the households at any given period; and (iii) ‘interhousehold differences in the intrahousehold change’ that indicate the differences in the growth process of food security situation observed by households over the months (Bollen & Curran, 2006; Curran et al., 2010; Grimm et al., 2017; Preacher, 2008). We have estimated these changes within the SEM framework. The SEM framework is relevant for the analysis of the underlying food security situation in our study as it offers flexible and robust estimation methods that are suitable for the analysis of the latent growth process in contrast to other competing methods (Bollen & Curran, 2006; Kline, 2011; Whiteman & Mroczek, 2007).

To capture the relevant features of the growth process of food security in our research area, we tested six growth models and selected the best model according to the statistical tests prescribed by the SEM literature. To that, we began with the estimation of the unconditional Single GCM as our base model to capture the overall growth trajectory of food security in our research area. Then, we estimated the conditional Single GCM accounting for the month-specific effect of the household’s primary occupation in FCS. Afterwards, we further expanded the model by incorporating the piecewise GCM in our analysis based on the theoretical and empirical evidence on seasonal variation of crop production and consumption. Lastly, we estimated the multi-group piecewise GCM at the cluster level conditioned on the household’s primary occupation as our final model, thereby, capturing the cluster-specific spatial heterogeneity in the food security situation in our research area.

We estimated the unconditional single GCM in the following form:

2 $$ {FCS}_{it}={\alpha}_i+{\lambda}_t{\beta}_i+{\varepsilon}_{it} $$

where *FCS*_*it*_ is the observed measure of food security, i.e., food consumption score for household *i* at month *t*, *α*_*i*_ is the model-implied latent intercept factor that indicates the status of food security in the reference month *t* such that *λ*_*t*_ = 0, *λ*_*t*_ represents the passage of time in terms of month, *β*_*i*_ is the latent slope-factor that quantifies the instantaneous rate at which the trajectory changes from one month to the next, and *ε*_*it*_ is the error term.

Then, we assessed the conditional GCM as follows:

3 $$ {FCS}_{it}={\alpha}_i+{\lambda}_t{\beta}_i+{\gamma}_{it}{Occupation}_{it}+{\varepsilon}_{it} $$

where, *γ*_*it*_ controls for the time-varying effect of the household’s primary occupation on food consumption score. The primary occupation refers to the choice of the household head to pursue either a subsistence-based agricultural production (*Occupation*_*it*_ = 1) or casual labor in floriculture sector (*Occupation*_*it*_ = 0). These two sectors of primary occupation reflect the distinct household economic structure, which, however, does not remain constant across the months due to seasonal variation in crop production. In that regard, *γ*_*it*_ controls for the effect of month-specific variation in the household’s economic structure in the estimation of the latent growth trajectory of food security.

The latent intercept and the latent slope factors estimated in eqs. (2) and (3) are referred to as ‘growth factors’ as they determine the direction and magnitude of the latent growth trajectory of food security (Flora, 2008). We estimated these factors as the following:

4 $$ {\alpha}_i={\mu}_{\alpha }+{\zeta}_{\alpha i} $$

5 $$ {\beta}_i={\mu}_{\beta }+{\zeta}_{\beta i} $$

where *μ*_*α*_ and *μ*_*β*_ are the means of the intercept and the slopes of the growth trajectories faced by each household *i* in the population. The mean of the intercept indicates the average food security situation at the reference month, and the means of the slopes estimate the growth rate or the rate of intrahousehold change in the latent process of food security. We also estimated variances of *μ*_*α*_ and *μ*_*β*_ as well as their covariances in eqs. (4) and (5). The variances of *μ*_*α*_ and *μ*_*β*_ capture the interhousehold heterogeneity in the initial status of the average food security situation at the reference month as well as in the rate of the intrahousehold change in the latent growth trajectory of food security, respectively. The covariances between *μ*_*α*_ and *μ*_*β*_ estimate the interrelationship between the initial status and the intrahousehold rate of change of food security. *ζ*_*αi*_ and *ζ*_*βi*_ are the disturbance terms that are expected to have zero means and are assumed to be unrelated with *ε*_*it*_.

We then proceeded with the estimation of piecewise GCM to capture any possible temporal heterogeneity in the growth trajectory of food security in our research area (Duncan et al., 2006; Sayer & Willett, 1998). Single GCM in eqs. (2) and (3) does not capture this aspect. However, it is imperative to note that even though we estimate different growth rates of food security in different periods in the piecewise GCM approach, the growth process in each of the analysed periods is still a linear combination of growth parameters (Preacher, 2008). For comparison, we have estimated both unconditional and conditional piecewise GCMs in our analysis as follows:

6 $$ {FCS}_{it}={\alpha}_i+{\lambda}_{1t}{\beta}_{1i}+{\lambda}_{2t}{\beta}_{2i}+{\lambda}_{3t}{\beta}_{3i}+{\varepsilon}_{it} $$

7 $$ {FCS}_{it}={\alpha}_i+{\lambda}_{1t}{\beta}_{1i}+{\lambda}_{2t}{\beta}_{2i}+{\lambda}_{3t}{\beta}_{3i}+{\gamma}_{it}{Occupation}_{it}+{\varepsilon}_{it} $$

where, eqs. (6) and (7) represent unconditional and conditional piecewise GCM, respectively, *β*_1*i*_, *β*_2*i*_ *and β*_3*i*_ are the latent slope factors in periods 1, 2 and 3, and *γ*_*it*_ controls for the month-specific effect of the household’s economic structure as proxied by the household’s primary occupation on FCS. Analogous to single GCM, we derived the estimate of the means and variances of the growth factors as well as their covariances in eq. (8), (9), (10) and (11) as follows:

8 $$ {\alpha}_i={\mu}_{\alpha }+{\zeta}_{\alpha i} $$

9 $$ {\beta}_{1i}={\mu}_{\beta 1}+{\zeta}_{\beta 1i} $$

10 $$ {\beta}_{2i}={\mu}_{\beta 2}+{\zeta}_{\beta 2i} $$

11 $$ {\beta}_{3i}={\mu}_{\beta 3}+{\zeta}_{\beta 3i} $$

Finally, we estimated the multi-group conditional piecewise GCM in eq. (7) in plantation and subsistence-agriculture clusters to investigate the spatial dimension of the growth trajectory of food security.

The literature on piecewise GCM suggests that the definition of transition points (knots) that categorises the growth process into distinct periods as in eqs. (6) and (7) should be based either on theory, previous studies, or empirical evidence in the sample (Flora, 2008). To identify potential seasonal dynamics influencing the trend of the FCS components in the sample, we selected the transition points referring to the crop calendar presented in Table 1 and the information on consumption collected by our questionnaire for the computation of FCS. We have set the knots in June and October to distinguish the following three periods in our analysis. According to our data and results of our FGDs, Period 1 refers to the months from March to June and is characterised by the hunger and rainy season when households primarily harvest and consume traditional crops and potatoes, and most importantly, consume the least diversified diet as compared to all other months. Period 2, between June and October, coincides with the peak-harvesting season of beans. During this period, households witness a gradual decline in the consumption of potatoes and diversify their diet towards beans and milk, and the number of households consuming beans shows a relatively higher increase if compared with the other food items. At the same time, the number of households consuming meat reduces because of a partial substitution effect with beans and milk. Finally, period 3 refers to months from October to January when the household availability and consumption of self-produced maize exceed the declining levels of beans’ production and consumption.

We evaluated the fit of our models based on the statistical tests suggested by the SEM literature (Curran et al., 2010; Preacher, 2008; Wang & Wang, 2020). More precisely, we tested our model against the optimal values for chi-square estimates, two incremental fit indices, namely, Comparative Fit Index (CFI) and Tucker Lewis Index (TLI), three parsimony indices, including Akaike’s information criterion (AIC), Bayesian information criterion (BIC), and Root Mean Square Error of Approximation (RMSEA), and an absolute fit index of Standardised Root Mean Square Residual (SRMR). Table 2 summarises the recommended optimal values for these tests.

**Table 2:** Goodness-of-fit indices

Fit type	Index	Interpretation
Absolute	SRMR	≤0.08: good fit
Parsimonious	RMSEA	≤0.06 and ≤ 0.08: good fit ≤0.05: very good fit
	AIC	Lower the value, better the fit
	BIC	Lower the value, better the fit
Incremental	CFI	≥0.90 and ≤ 0.94: good fit ≥0.95: very good fit
	TLI	

SRMR: Standardised Root Mean Square Residual, RMSEA: Root Mean Square Error of Approximation, AIC: Akaike’s information criterion, BIC: Bayesian information criterion, CFI: Comparative Fit Index, TLI: Tucker Lewis Index

Source: Adapted from Gana and Broc (2019, p. 43) and Wang and Wang (2020)

It is important to consider that there are no statistical gold standards for model selection in SEM, and the literature features an array of fit indices that are, however, still being developed over the years (Kline, 2011). Nevertheless, the literature suggests following the thresholds mentioned above as an indicative guideline for model evaluation, as also highlighted by Hu and Bentler (1999) and Schreiber et al. (2006).

## Results

Table 3 shows the measure of goodness-of-fit for our six models. It highlights that the unconditional single GCMs offer relatively weaker model fit than their corresponding three-piece GCMs as indicated by their respective AIC and BIC. The three-piece GCMs offer an acceptable range of values for absolute (SRMR), parsimonious (RMSEA), and incremental (CFI and TLI) fit indices.

**Table 3:** Evaluation of Model Goodness-of-Fit

Estimated Models	Fit-Indices						
	AIC	BIC	Chi-square	SRMR	RMSEA	CFI	TLI
Single GCM							
Unconditional model	51,357.98	51,428.36	404.81	0.10	0.10	0.89	0.90
Conditional Model (with Occupation)	51,265.78	51,384.54	508.68	0.06	0.06	0.90	0.90
Multi-Group Model (conditioned on Occupation)	51,271.30	51,508.83	762.73	0.07	0.06	0.88	0.88
Three-piece GCM							
Unconditional model	51,283.41	51,393.37	315.83	0.08	0.09	0.91	0.91
Conditional Model (with Occupation)	51,191.91	51,350.26	417.69	0.05	0.05	0.92	0.92
Multi-Group Model (conditioned on Occupation)	51,178.63	51,495.33	630.71	0.06	0.05	0.91	0.90

Source: Authors’ elaboration based on primary sources of data

The relatively better statistical strength of piecewise GCM in explaining the observed variation in our sample corroborates the theoretical and empirical consideration that we applied in our analysis in categorising the longitudinal sample into three distinct time periods with the knots set in June and October. Table 4 presents the results of all GCMs to provide a comparative analysis of the food security situation in our research area.

**Table 4:** Growth Curve Model Estimates with Intercepts Fixed in June

Features of Growth Trajectory		Estimated Model			
		Unconditional Model	Conditional Model	Multi-group Model (Conditioned on Occupation)	
Growth factor	Parameter			Plantation Cluster	Subsistence-Agricultural Cluster
Single GCM					
Intercept	Mean	73.55***	73.35***	72.55***	74.35***
	Variance	98.51***	98.75***	92.82***	102.67***
Slope	Mean	0.88***	0.85***	0.99***	0.65***
	Variance	0.72***	0.74***	0.59***	0.9***
	*Covariance*	0.59	0.44	1.13	−0.03
Three-piece GCM					
Intercept	Mean	73.25***	73.32***	72.53***	74.22***
	Variance	129.56***	129.31***	117.3***	142.59***
Period 1					
Slope	Mean	0.73***	0.91**	1.23***	0.38
	Variance	8.52***	8.72***	1.38*	14.65***
	*Covariance*	19.94***	19.65***	11.57**	25.82***
Period 2					
Slope	Mean	1.09***	0.98***	1.28***	0.61**
	Variance	1.43**	1.46**	1.41*	1.56*
	*Covariance*	−3.08	−3.12	−2.02	−4.27
Period 3					
Slope	Mean	0.5**	0.52*	0.2	0.86**
	Variance	2.95*	3.46**	4.81**	2.55
	*Covariance*	−5.17*	−5.28*	−2.28	−8.23**

Source: Authors’ elaboration based on primary sources of data

Notes: *Covariance* refers to the covariance between the intercept and the slope. Covariance between slopes in the three periods are statistically insignificant and are not reported in the table. *** p value <0.001; ** p value <0.01; * p value <0.05

The results in Table 4 are from the models estimated with the intercept term (*α*_*i*_) fixed in June. The month indicating the intercept serves as the reference point, or baseline, against which we interpret the growth estimates. The literature recommends that the choice of the reference month should not be arbitrary (Preacher, 2008) and should be motivated by the underlying meaning of the food security situation at that particular time point. Based on our data, we have chosen to present the results of GCMs with an intercept fixed in June to underline a meaningful analysis of household’s food security situation in reference to the period when a large number of households introduce a new category of a food item (beans) in their consumption as well as production. Nevertheless, we also estimated all the models with intercepts fixed in each of the 11 months, which confrmed the results that we obtained by setting the intercept in June.

### Intrahousehold change in food security trajectory

**Fig. 1:**
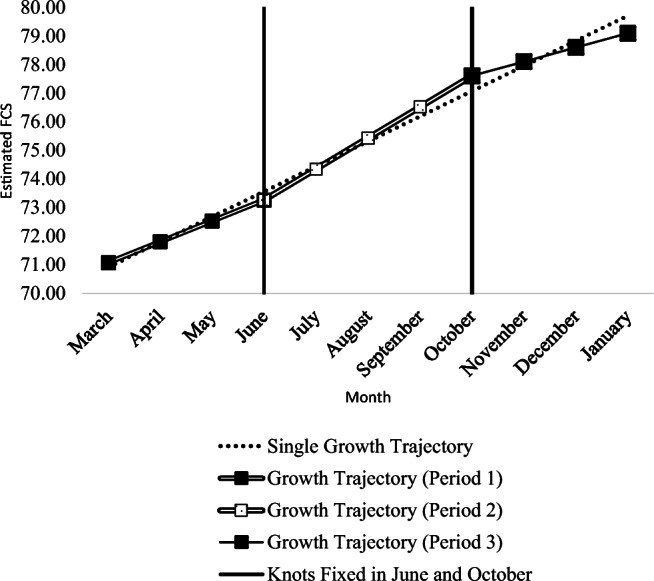
Comparison of Unconditional Single and Piecewise GCMs.

The results of the unconditional single GCM (in Table 4) indicate a statistically significant improvement in the food security situation for households in our research area over the studied months (from March 2018 to January 2019). However, once we accounted for seasonality in three-piece GCM, we observe that the uniform growth rates estimated by the single GCM are not applicable across these months. Statistically, we established the relevance of the three-piece GCM over single GCM by running a likelihood ratio test for model-comparison imposing equality constraints on latent means, variances, and their covariances in piecewise GCM. The significant difference in chi-square value (${\upchi}_{\mathrm{df}(9)}^2$ = 92.57, *p* value <0.001) confirmed the relevance of the three-piece GCM over single GCM. Therefore, our evidence confirms the presence of temporal heterogeneity in the intrahousehold growth rate of the food security situation in our research area, as also highlighted in Fig. 1. The figure compares the latent growth process in food security estimated by the unconditional single and three-piece GCMs, as presented in Table 4.

In Fig. 1 the uniform growth rate estimated by single GCM either under- or overestimates the trajectory of the food security situation in our research area. Focusing on the unconditional three-piece GCM, the improvement in food security is relatively higher in period 2 as compared to other periods. More precisely, the growth rate of latent food security in period 2 was 1.49 and 2.18 times the growth rates observed in period 1 and period 3, respectively. This temporal heterogeneity in the growth rates of food security remained unchanged even after accounting for the possible impact of households’ occupational choice on FCS.

**Fig. 2:**
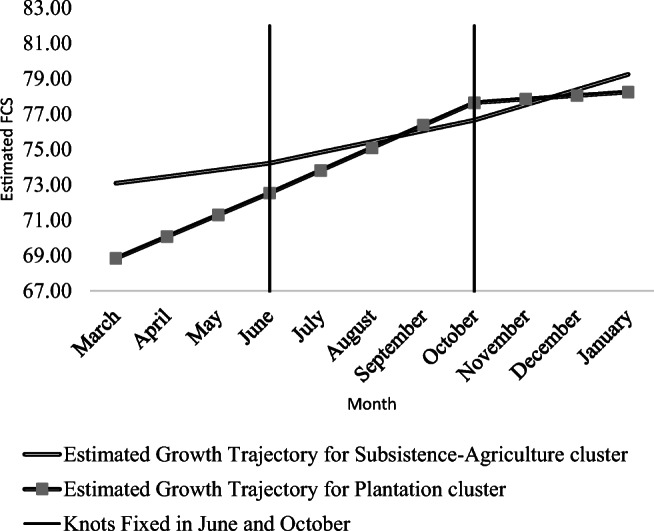
Comparison of Multi-Group Piecewise GCM Conditioned on Occupation.

The multi-group analysis conducted at the cluster-level revealed additional important specificities in the subsistence-agricultural and plantation clusters. Figure 2 illustrates the latent growth process of the food security situation in the two clusters, as predicted by the estimated growth factors for multi-group three-piece GCM presented in Table 4.

In the subsistence-agricultural cluster, households, on average, do not witness significant improvement in their food security status during the hunger season, as suggested by the statistically insignificant estimated slope of latent growth trajectory (0.38, *p* value >0.05) corresponding to period 1. This growth rate, however, becomes positive and statistically significant in period 2 (0.61, p value<0.01) and proceeds at a higher rate in period 3 (0.86, p value<0.01) relative to the household food security situation in June. This increase in the growth rates in the second and the third periods is in parallel with the gradually increasing production, partial consumption, and the sale of food items by smallholder farmers post June and October in the subsistence-agricultural cluster. As shown by Sassi (2019), the second period witnesses the harvesting peak of beans and potatoes as well as the beginning of the maize harvesting season that reaches its apex in the third period.

Based on our data and FGDs, Beans, potatoes, and maize are the three most important food items consumed in terms of frequency and nutrient density by the dominant smallholder farmers in the subsistence-agricultural cluster. Therefore, our results confirm, at least, partially, the relationship between agricultural production and diet diversification and subsequent improvement in food security, as also highlighted by many in the literature (e.g., Aweke et al., 2020; Pellegrini & Tasciotti, 2014; Romeo et al., 2016; Sassi, 2019; Sibhatu et al., 2015). Moreover, during the third period, smallholder farmers commercialise a large part of their produced maize, which might subsequently improve their access to other foods, thereby contributing to the increased growth rate of the food security situation as indicated by the FCS.

In contrast, a different situation characterises the plantation cluster where the agricultural season seems to have relatively less effect on the growth trajectory of the household food security situation as compared to that in the subsistence-agricultural cluster. Nevertheless, Fig. 2 highlights that the predicted growth trajectory of food security is relatively at lower levels in the plantation cluster in comparison to the other cluster in most of the months. As noted by Sassi (2019), these lower levels of food security in the plantation sector are likely to be, in part, explained by a low-income situation that affects a large part of the population, and subsequent limited access to food. Most households in the plantation cluster purchase the food they consume, which is subject to local market price conditions. High food prices during the hunger season (period 1), in conjunction with a lower compulsory minimum wage, which were reported to be below the food poverty line during focus group discussions, adversely affect their food security situation throughout.

Despite the lower levels of food security in March as compared to June, we witnessed a sharp increase in the growth process during the first (1.23, *p* value <0.001) and the second periods (1.28, p value <0.001) in the plantation cluster. These growth rates in the plantation cluster in the first and the second periods were 3.23 and 2.09 times the growth rates observed during the same period in the subsistence-agricultural cluster, respectively. In the third period, the growth rate ceased to improve, as indicated by the statistically insignificant estimated slope (0.2, p value>0.05). Despite this, the accelerated growth rate in the plantation cluster produced a convergence of growth trajectories in the two clusters in the middle of the second period leading to a higher level of food security than that in the subsistence-agricultural cluster for two months up to the third period. Therefore, we observed that even with the stagnation of growth rate in the third period, the estimated food security level, on average, was at an acceptable level for all the households in our sample living in the plantation cluster, relative to their status in June.

### Interhousehold heterogeneity in food security trajectory

The analysis of the variances and covariances of the intercept and the slope of the latent growth process provides interesting new elements in the dynamics of the food security situation. We found a substantial degree of temporal and spatial interhousehold heterogeneity in the manner households witness changes in their food security levels in our research area. Our analysis identified two different typologies of heterogeneity in the food security trajectory.

The first type of heterogeneity relates to the interhousehold heterogeneity in the manner households witness change in their food security levels, as measured by the variance of the growth rate estimates (slope) of piecewise GCM in Table 4. In the overall investigated area, the heterogeneity of growth rates was higher in period 1 (8.52, *p* value<0.001), declined in period 2 (1.43, p value <0.01), and increased in period 3 (2.95, p value <0.05). These findings remained intact even after controlling for the effect of the occupational choice of the household head.

The cluster-level piecewise GCM highlights that the interhousehold heterogeneity in the growth rates was primarily concentrated during the hunger season in the subsistence-agricultural cluster (14.65, p value <0.001 in period 1). These results possibly indicate that agricultural production and diet diversification not only relate to the subsequent improvement in food security, as previously highlighted, but also contribute to the heterogeneous impact on such improvement. The variance of the slope imply whether food security situations are different across households. The statistical insignificance of the variance of the slope in period 3 in the subsistence-agriculture cluster (2.55, p value >0.05) suggests that a better access to food results into a similar level of improvement in the food security situation for our sample households. On the other hand, the significant and higher variance of growth rate in the third period in the plantation cluster reveals the possibility that not all households attain acceptable levels of food security.

The second type of heterogeneity relates to the covariance between the food security situation in the reference month and the growth rates of food security in the three periods. In other words, the covariance between the intercept and the slope estimates in the three periods provides a dynamic picture of inequalities in the improvement of food security over the months. Estimates of covariances between the intercept and the growth rates for unconditional piecewise GCM in Table 4 strongly suggest that the inequality in the food security improvement is both time and cluster-specific. In the overall area, households starting at higher (or lower) levels of food security in March witnessed higher (or lower) levels of food security in June, thus significantly widening the inequality in the food security improvement in period 1 (covariance of Intercept and Slope in Period 1 = 19.94, *p* value <0.001). The interhousehold differences in food security improvement remained constant during period 2 (covariance of Intercept and Slope in Period 2 = −3.08, p value >0.05) for all households. In period 3, the inequality in the food security improvement, relative to June, began to decline (covariance of Intercept and Slope in Period 3 = −5.17, *p* value <0.001). However, such a declining effect witnessed between June and January was 3.86 times less than the widening effect witnessed in Period 1. These conclusions remained intact even after controlling for the occupational choice of the household head.

A cluster-level analysis of piecewise GCM further reveals the significant spatial heterogeneity in the dynamics of unequal food security improvement over the months. We observed that the increase in the inequality of food security improvement in period 1, as suggested by the unconditional model, was more accentuated in the subsistence-agricultural cluster than in the plantation cluster during the hunger season. Though households in the subsistence-agricultural cluster witness a significant decline in the inequality in period 3, such a declining effect was 3.14 times less than the widening of inequality in period 1. In the plantation cluster, the dynamics of inequality is more worrisome. The inequality in food security improvement witnessed in period 1 (covariance of Intercept and Slope in Period 1 = 11.57, p value <0.01) did not decline in the succeeding two periods, despite an overall improvement in food security levels. This high level of inequality was also reflected in the significant higher variability of growth factors in the plantation cluster in period 3.

## Discussion

Our paper highlights the complexity of the food security mosaic around the Lake Naivasha Basin, and stresses the importance of considering both the severity of food insecurity and also its temporal and spatial dynamics, especially accounting for the seasonality in the agricultural production process and cluster specificities. The inability to consider these aspects might lead to an ineffectual design and implementation of interventions for different categories of food insecure households. The results of our analysis strongly assert that the seasonal dynamics of heterogeneous food security levels matter.

The discussion of a seasonal effect on food security is common in the literature. In particular, the literature highlights cyclical fluctuations in access to food, employment and income for smallholders working on family farms in developing countries (Berton et al., 2013; Ferro-Luzzi et al., 2002; Hesselberg & Yaro, 2006; Hirvonen et al., 2016; Sassi, 2012, 2015). This situation is also observed in Kenya where smallholder farmers are subjected to the seasonal variability in the rainfall that determines their crop production, as suggested by Table 1. Therefore, their food and income depend on their annual or semi-annual harvest. Moreover, the price of their produce normally decreases after harvest and peaks in the lean season, as evident during our FGDs. For this reason, food insecurity and malnutrition increase in the lean season, especially for rural population confirming the literature focused on research areas similar to ours (Brander et al., 2021; Sassi, 2019).

Moreover, the literature also offers a comparative analysis of the food security situation between the pre- and post-harvest seasons (Ayenew et al., 2018; Becquey et al., 2012). Our paper contributes two new aspects to this literature. First, it compares the seasonal dynamics of food security in areas characterised by two different dominant agricultural sectors: subsistence-agriculture sector and the commercial plantation sector, thereby contributing to the policy debate on the role of commercial agriculture on achieving food security. Second, it provides a descriptive analysis of the distributional consequences of such seasonal dynamics, accounting for both temporal and spatial heterogeneity in the improvement of the food security situation.

### Role of commercial agriculture in achieving food security

The role of cash-crops in achieving food security is one of the central topics in the ongoing food policy debates in Sub-Saharan Africa (Kuma et al., 2019), however, with inconclusive and mixed empirical evidence (Tankari, 2017). To that, our specific study offers a comparative overview of the food security situation in the predominantly cash-crop based plantation cluster with respect to the subsistence-agricultural cluster. As shown in Fig. 2, our results highlight that the household food security is lower in the plantation cluster than in the subsistence-agricultural cluster during the hunger season. The same situation characterizes the first harvesting period of food crops (beans, potatoes, and maize) that are important from a nutritious point of view. Despite such lower levels, our evidence indicates that the food security level improves faster in the plantation cluster during that period. These results indicate a positive contribution of commercial agriculture in the improvement of the food security situation during the hunger season in the Basin during the months from March to June confirming the study by Sassi (2019).

Further, the growth trajectory of food security in the subsistence-agricultural cluster in the third period also seems to confirm the positive impact of commercial agriculture on food security. This period coincides with the harvesting season for maize, which is the dominant staple food in much of Kenya. Maize is one of the major food crops grown in our investigated area, where it also acts as a cash crop in specific periods, especially during the post-harvest season. Farmers sell a large part of their maize produce to traders in the post-harvest period, irrespective of their farm size and despite the lower selling price due to higher levels of supply, as reported during FGDs. Therefore, maize stands as an important source of income, especially for smallholder farmers, which consequently improves their access to food in the market, as confirmed during FGDs. Based on these results, our study supports the body of literature that indicates specialisation in cash-crop production (maize in our case) as a means to increase the income of farm households, reduce rural poverty and achieve a higher level of welfare, including food security (Govereh & Jayne, 2003; Poulton et al., 2001; Timmer, 1988).

However, our study suggests a cautious interpretation of the positive role of commercial agriculture as we find that such a positive effect might only be applicable in the short run. This limitation of commercial agriculture was evident during FGDs and key-informant interviews. Our qualitative evidence reveals that most traders in the investigated area do not sell maize in the domestic market, thereby subsequently raising the local market price for maize, and consequently, limiting the access to purchased food. Besides, the evidence from our FGDs indicate that smallholder farmers store only a part of their maize produce that, however, does not last for the entire year. Consequently, households experience lower levels of food security in the successive periods, as also witnessed in period 1 in our study.

### Distributional consequences of seasonal dynamics in food security

Our analysis indicates that the improvement of food security is unequal in both the clusters in our sample. In Sub-Saharan Africa, attention on inequality has risen substantially with the introduction of the Sustainable Development Goals. The international community agrees that addressing income disparities in the region is a fundamental step to reaching the objectives set by Agenda 2030 (Odusola et al., 2017). Our study highlights that the attention on tackling inequality should also incorporate disparities in food security. More importantly, our evidence shows that the problem of food security inequalities has links with both seasonal and cluster-specific economic structures, which we explain below.

Inequalities in the improvement of food security primarily manifest during the hunger season in our study area. Such inequalities were not only confined to the lesser developed areas such as the subsistence-agricultural cluster we studied, but are also substantial in the plantation cluster where farming practices are capital intensive. Therefore, our paper suggests that technological progress alone might not be sufficient to promote an equal food distributional process when poverty and unequal development remain relevant, as also discussed in other studies (see Go et al., 2007). Almost 40% of the households in the plantation cluster in our sample are below the poverty line. Besides, the average levels of education in our sample was relatively low, which acts as an access barrier to better and lucrative jobs. During our FGDs, it was also evident that economic access to food was the major constraint for the sampled households, especially during the hunger season when households face high food prices combined with low income. This situation is confirmed by the literature on Sub-Saharan Africa (Simtowe & De Groote, 2021). We also noted that household members in the plantation cluster, particularly in the most vulnerable households, compensate their low income by engaging in activities in the informal or subsistence agriculture, thereby making a strategic switch in their occupational choice to cope up with lower levels of food security. This highlights the important role of subsistence agriculture, even in the context of the commercial plantation sector, for mitigating food insecurity.

In the subsistence-agriculture cluster, the increase in the inequality of improvements in food security in the first period, and the subsequent decline in the third period seem to be linked with the production of maize and with income levels. More specifically, the decrease in inequality in the third period indicates a possible equalising effect of maize production and subsequent increase in its consumption and any excess sale. However, as also discussed previously, the structural issues related to the supply of maize by local traders, lower returns for farmers in the sale of their produce, possible price increases in the local market, and inability to store the grain over a long period of time in the third period seem to adversely impact the distribution of food security improvements for households in the subsequent periods, as also evident in higher estimates of inequality in the first period. This result suggests a potential positive role of improving market structure and storage infrastructure in tackling the food security disparities in the subsistence-agriculture cluster in the Lake Naivasha Basin.

## Conclusion

In this paper, we employed a novel approach to analyzing food security as a latent concept through the adoption of a multi-group piecewise latent trajectory model within a robust Structural Equation Modelling framework. We presented a novel investigation of the food security considering it as a latent variable. For that purpose, we capitalized on the monthly primary panel data that we collected in rural Kenya. Our analysis confirmed the presence of household-level heterogeneities in food security outcomes, as discussed in much of the literature. It also advanced new evidence on the dynamics of such heterogeneities. It is imperative to distinguish the results generated by previous traditional longitudinal analysis and our study. The former has contributed to the understanding of an average change in food security levels and at most, the inter- and intrahousehold heterogeneities in food security in a specific period. Our advancement concerned the question of how such inter- and intrahousehold heterogeneities vary across the months in different seasons and in different clusters of farming households, while also accounting for the occupational disposition of the household head. Our study has offered significant evidence on the dynamic nature of inequalities in household-level food security improvements in the specific context of subsistence and commercial agricultural clusters in Kenya. This evidence is vital for policymakers to identify a better targeting period for food security interventions considering both seasonal and cluster specificities of the targeted population.

Our study highlights the complementary role of subsistence and commercial agricultural sectors and suggests the incorporation of the subsistence agricultural sector and the informal economy in the discussion of commercial agriculture for food security policies. Our analysis highlights the need for policy interventions to help subsistence farmers to store and market their surplus of commercial produce, especially maize, in their transition to the commercial agricultural system, which could then help them avoid adverse effects of seasonal patterns of crop production on household consumption, balanced dietary intake, and optimal nutrition. Further, our results make a strong case for the need of multidemensional approach to food security that promotes inclusive agricultural development and income inequality reduction policies to tackle food security heterogeneities among households.

Our study, however, is based on the assumption of ‘selection on observables’, which implies that we do not negate the possibility that some other factors not observed in our models could also explain food security in our research area. Given that assumption, our study did not intend to determine the causal factors behind the seasonal pattern of heterogeneous food security growth trajectories and subsequent temporal and spatial inequalities in food security. Therefore, future studies will be needed to determine causal factors behind the within household growth of food security in different periods and clusters. In this regard, introducing time varying covariates could provide more robust estimates of the latent growth process of food security and its causal determinants.

## Appendix

**Table 5:** Descriptive statistics of the Food Consumption Score

Month	Overall sample		Subsistence-agricultural cluster		Plantation cluster	
	Mean	SD	Mean	SD	Mean	SD
March	70.16	17.43	70.64	16.32	69.83	18.15
April	73.83	14.49	73.31	14.43	74.18	14.54
May	70.30	12.98	69.50	13.45	70.85	12.64
June	73.04	14.51	72.23	13.94	73.59	14.87
July	75.65	14.25	75.85	14.16	75.52	14.33
August	76.13	15.23	75.43	16.03	76.60	14.66
September	74.44	14.73	74.41	14.48	74.47	14.91
October	78.00	13.94	78.40	13.37	77.73	14.33
November	76.87	14.30	76.30	14.19	77.25	14.38
December	82.48	14.27	84.11	13.83	81.37	14.47
January	77.67	14.63	77.06	14.72	78.09	14.58
*n*	601		243		358	

Source: Authors’ elaboration based on primary sources of data

Notes: n = number of observations, SD = Standard deviation of FCS
